# Investigating the Glucagon Receptor and Glucagon-Like Peptide 1 Receptor Activity of Oxyntomodulin-Like Analogues in Male Wistar Rats

**DOI:** 10.1016/j.curtheres.2015.10.003

**Published:** 2015-11-02

**Authors:** Samantha L. Price, James S. Minnion, Stephen R. Bloom

**Affiliations:** Department of Investigative Medicine, Imperial College London, London, United Kingdom

**Keywords:** analogue, body weight, glucagons, oxyntomodulin

## Abstract

**Aims:**

To investigate the effect of Glu-3 OXM-like analogues on food intake and bodyweight in male rats.

**Background:**

Oxyntomodulin (OXM) is a natural agonist at both the glucagon receptor (GCGr) and the glucagon-like peptide 1 receptor (GLP-1r), and peripheral administration reduces food intake and increases energy expenditure in rodents and humans. Substituting the native glutamine (Gln) at amino acid position 3 of OXM for glutamate (Glu) has previously been shown to diminish GCGr activity without affecting GLP-1r activity. The effects of Glu-3 OXM analogues have not been investigated in rats.

**Methods:**

The effect of 2 Glu-3-substituted OXM-like analogues (eg, OXM14E3 and OXM15E3) on food intake and body weight was investigated in male Wistar rats during 6 days of daily subcutaneous (SC) administration. The effects of Glu-3 substitution on analogue binding and activity at the rat GCGr and rat GLP-1 receptor were investigated in vitro using Chinese hamster ovary or Chinese hamster lung cells.

**Results:**

We report the novel finding that 2 5-nmol/kg Glu-3 OXM-like analogues (OXM14E3 and OXM15E3) significantly increased rat body weight by up to 4% compared with the equivalent non-Glu-3 analogues (OXM14 and OXM15), without affecting food intake. The effect of OXM15E3 on body weight was dose–dependent. Glu-3 analogues, including Glu-3 OXM, decreased glucagon-mediated cyclic adenosine monophosphate accumulation in Chinese hamster ovary cells expressing the rat GCGr, suggesting they may be acting as antagonists.

**Conclusions:**

The results indicate Glu-3 OXM-like analogues might not be suitable tools to investigate the mechanism of OXM analogue action in a rat model because they significantly increase body weight independent of food intake. Glu-3 OXM analogues are partial agonists at the rat GCGr and may also act as antagonists, possibly resulting in the observed increase in body weight.

## Introduction

The gut-derived hormone oxyntomodulin (OXM) is a naturally occurring dual agonist of both the glucagon receptor (GCGr) and glucagons-like peptide 1 receptor (GLP-1r).^1^ Structurally OXM is the 29 amino acids of glucagon with a C-terminal octapeptide tail.^2^ Administration of OXM to rodents and humans reduces food intake and increases energy expenditure, generating significant weight loss and highlighting OXM as a potential pharmacologic treatment for obesity.^3^, ^4^ Due to the short in vivo half-life of native OXM,^5^ it is necessary to produce long-lasting analogues for clinical use.

The dual agonist properties of OXM make investigating its mechanism(s) of action difficult. Using GCGr or GLP-1r antagonists can interfere with the actions of endogenous glucagon and GLP-1, hormones that play critical roles in glucose homeostasis, giving an inaccurate picture of OXM actions. Alternatively, substituting glutamine at position 3 of OXM with Glu has previously been reported to diminish GCGr activity without affecting GLP-1r activity,^6^, ^7^ enabling investigation of mechanisms of action relating to exogenous OXM administration.

These Glu-3 OXM analogues have been used in mice to investigate the contribution of GCGr and GLP-1r activity to the function of both native OXM^7^, ^8^ and a derivatized analogue.^6^ Previous results indicate that OXM activity at the GLP-1r is responsible for the reduction in food intake and activity at the GCGr increases energy expenditure.^6^, ^7^

Previous work in our lab designed OXM-like dual analogues of the GCGr and GLP-1r based on the 29 amino acids of glucagon. A number of conservative amino acid substitutions were made to increase GLP-1r activity, improve peptide stability, and extended circulatory half-life, with a view to developing a potential treatment for obesity. Several equivalent Glu-3 analogues were also designed to investigate the contribution of GCGr and GLP-1r activity to the effects of the OXM-like dual analogues. Our pilot experiments suggested that Glu-3 OXM-like analogues increased body weight in rats without affecting food intake; therefore, further experiments were undertaken to clarify these findings. The 2 Glu-3 analogues OXM14E3 and OXM15E3 were chosen for investigation because they increased body weight in a pilot experiment and had similar conservative amino acid substitutions and pharmacokinetic profiles.

The results of our study suggest that Glu-3-substituted OXM analogues may be partial agonists that can act as competitive antagonists in vitro and increase body weight independent of food intake in vivo. Therefore Glu-3 OXM analogues are not a suitable tool to investigate the effects of OXM-like dual analogues in a rat model.

## Methods

### Compounds

**Figure 1:**
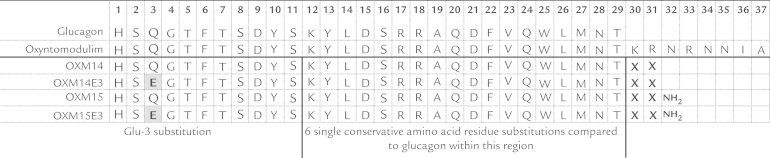
Schematic of the amino acid structures of the oxyntomodulin (OXM)-like dual analogues investigated in the study. Glu-3 = glutamate substitution at amino acid position 3.

All peptides (human glucagon, GLP-1, and OXM; analogues OXM14 and OXM15 and their respective Glu-3 analogues OXM14E3 and OXM15E3; and OXME3) were custom synthesized by Insight Biotechnology Limited (Middlesex, United Kingdom) and were >90% pure. The structure of analogues OXM14E3 and OXM15E3 were based on the 29 amino acids of glucagon and, other than the Glu-3 substitution, contained 6 single amino acid substitutions between residues 12 through 29. Both analogues had additional amino acids at residues 30 and 31 and OXM15E3 was C-terminally amidated (Figure 1). These analogues were chosen for investigation because they had similar pharmacokinetic profiles but different affinity and potency at the rat glucagon and GLP-1rs.

### Animals

Male Wistar rats (Charles River, Margate, United Kingdom) were maintained in individual cages under controlled temperature (21°C–23°C) and light conditions (12:12 light-dark cycle, lights on at 0700 hours) with ad libitum access to food (RM1 diet; SDS, Witham, United Kingdom) and water unless otherwise stated. All animal procedures were approved under the British Home Office Animals (Scientific Procedures) Act 1986 (Project Licence 70/7236).

### In vivo food intake and body weight studies

Before study commencement, rats were randomized to treatment groups with equal mean body weights. An injection of peptide (maximum volume 50 µL, reconstituted in zinc chloride to enhance peptide depot formation^9^, ^10^) or vehicle (saline) was administered subcutaneously at 1600 hours daily and food and body weight recorded.

### Cell culture

All cell culture reagents were obtained from Invitrogen (Life Technologies Ltd, Paisley, United Kingdom) unless otherwise stated. Chinese hamster ovary-K1 cells overexpressing the rat GCGr were routinely maintained in Dulbecco’s modified eagle medium (DMEM) with 25 mM glucose and 1 mM sodium pyruvate supplemented with 10% fetal bovine serum, 25 mM hydroxyethyl piperazineethanesulfonic acid, 1% antibiotic (100 U/mL penicillin and 100 µg/mL streptomycin), and 1 × nonessential amino acids. Chinese hamster lung cells overexpressing the rat GLP-1r were maintained in the same DMEM but without additional hydroxyethyl piperazineethanesulfonic acid or nonessential amino acids.

### In vitro cyclic adenosine monophosphate (cAMP) accumulation

Levels of cAMP accumulation was measured in Chinese hamster ovary-K1 cells overexpressing the rat GCGr (cDNA from Origene, Cambridge Bioscience, Cambridge, United Kingdom) or Chinese hamster lung cells overexpressing the rat GLP-1r (a gift from Bernard Thorens, University of Lausanne, Switzerland).

Cells were seeded in a 48-well plate (Nunc, VWR International Inc, Chicago, Illinois) in their respective supplemented DMEM, at a density of 37,500 cells/well (250 µL/well), then incubated for 24 hours. Cells were serum starved in DMEM +1% antibiotic for 1 hour before 30 minutes’ incubation at room temperature with peptide concentrations diluted in serum-free DMEM with 1 mM IBMX (Sigma-Aldrich, Dorset, United Kingdom). Each peptide concentration was applied in duplicate or triplicate per experiment.

For rat GCGr antagonism studies, cells were incubated for 30 minutes with peptide concentrations diluted in serum-free DMEM with 1 mM IBMX and 0.1 nM glucagon. Five wells of each concentration and 12 wells of 0.1 nM glucagon alone were applied per experiment.

Postincubation, medium was removed and cells lysed with 120 µL/well of 0.1 M hydrochloride + 0.5% nonionic, octylphenol ethoxylate surfactant. cAMP levels were measured using an indirect ELISA according to the manufacturer’s instructions (ADI-900-066, Enzo Life Sciences, United Kingdom), optical density was read at 405 nm on a Biotek ELx808 (Wolf Laboratories, York, United Kingdom). Data were plotted and in vivo efficacy values calculated.

### In vitro receptor binding: Supplementary

Refer to O’Shea et al^11^ for detailed methods. Briefly, membranes were prepared from Chinese hamster ovary-K1 cells overexpressing the rat GCGr or Chinese hamster lung cells overexpressing the rat GLP-1r by osmotic lysis and differential centrifugation. Membranes (100 µg protein) were incubated in siliconized polypropylene tubes with 100 pm (500 Bq) either iodine^125^ labeled GLP-1 or iodine^125^ labeled glucagon in competition with unlabeled peptide. Total specific binding was calculated as the difference in counts between assays in the presence (nonspecific) and absence (total) of up to 6000 nM peptides. Data were plotted and in vitro affinity values calculated.

### Statistical analysis

cAMP in vitro efficacy and binding in vitro affinity were calculated using nonlinear regression with Prism version 5 software (GraphPad Software Inc, San Diego, California). Differences in cumulative food intake and body weight were analyzed using 1-way ANOVA and post hoc tests with Dunnett or Bonferroni correction (Prism version 5 software). In all cases *P* < 0.05 was considered statistically significant.

## Results

### In vivo

**Figure 2:**
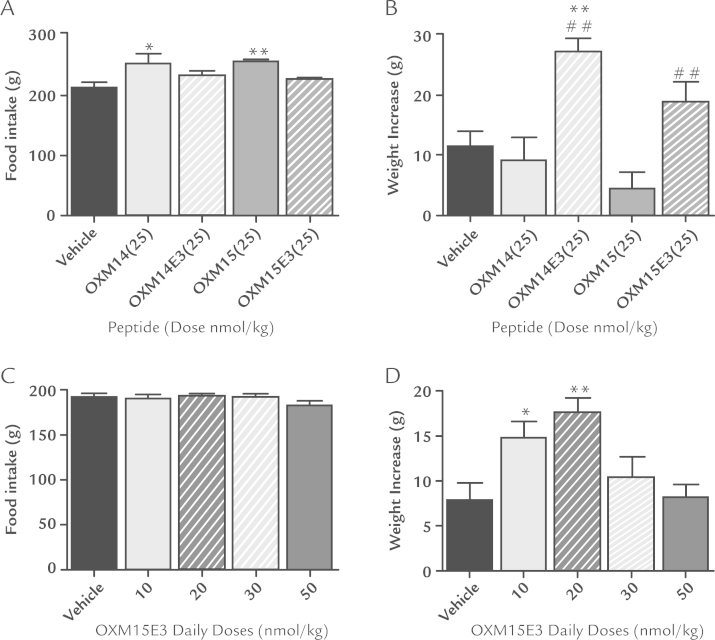
(A) Food intake and (B) body weight change after 7 days of daily subcutaneous (SC) 25 nmol/kg oxyntomodulin (OXM)14, OXM15, OXM14E3, OXM15E3, or vehicle (n = 6–7). (C) Food intake and (D) body weight change after 7 days of daily SC administration of various doses of OXM15E3 or vehicle (n = 11–12). Data are presented as mean (SEM). Statistical analysis was conducted using 1-way ANOVA and post hoc tests with Bonferroni (A and B) or Dunnett (C and D) corrections. *P < 0.05; **P < 0.01 versus vehicle controls; ^##^*P* < 0.01 versus peptide with native glutamine at amino acid position 3.

Daily administration of 25 nmol/kg OXM14E3 to rats significantly increased body weight over 7 days (27.5 [2.2] g; *P* < 0.01) compared with vehicle controls (11.4 [2.6] g) and OXM14 (9.2 [3.8] g) (Figure 2B). Daily 25 nmol/kg OXM15E3 administration significantly increased body weight over 7 days (19 [3.3] g; *P* < 0.01) compared with OXM15 (4.5 [2.7] g) (Figure 2B). Food intake was not increased after administration of either OXM14E3 or OXM15E3 (Figure 2A).

Daily administration of 10 nmol/kg and 20 nmol/kg OXM15E3 resulted in a significant increase in rat body weight over 7 days (15.1 [1.9] g; *P* < 0.05 and 17.7 [1.5] g; *P* < 0.01, respectively) compared with vehicle controls (7.9 [2.0] g) (Figure 2D), without altering food intake (Figure 2C).

### In vitro

**Figure 3:**
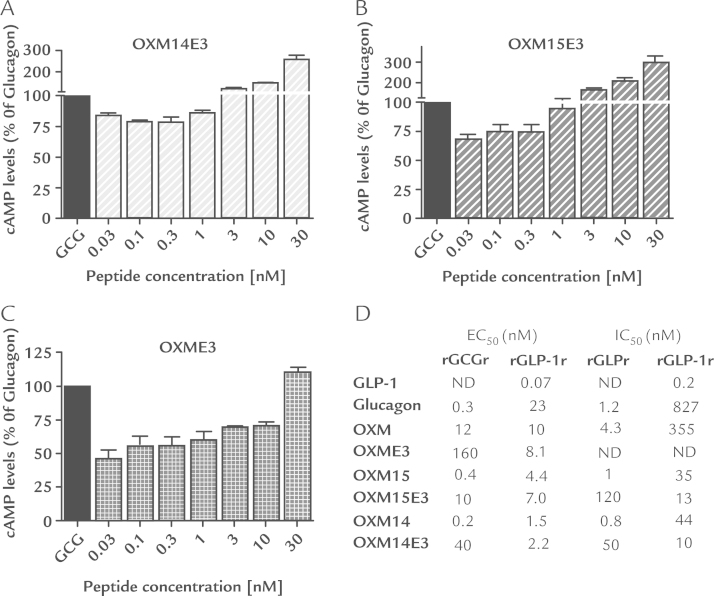
In vitro cyclic adenosine monophosphate (cAMP) levels measured in Chinese hamster ovary cells overexpressing the rat glucagon receptor (rGCGr) following incubation with various concentrations of (A) oxyntomodulin (OXM)14E3, (B) OXM15E3, or (C) OXME3 in the presence of 0.1 nM glucagon. Mean cAMP levels (SEM) are shown as a percentage of the levels produced by glucagon stimulation alone (n = 3). (D) Mean in vitro efficacy (cAMP EC50) and affinity (IC50) of OXM analogues and native peptides at the rGCGr and rat glucagons-like peptide 1 receptor (rGLP-1r). cAMP EC50 values were calculated using Chinese hamster ovary cells overexpressing the rGCGr or Chinese hamster lung cells overexpressing the rGLP-1r. IC50 values were calculated using membranes produced from the Chinese hamster ovary cells or Chinese hamster lung cells (n = 3).

At the rat GCGr the binding affinity of the Glu-3 substituted peptides OXM14E3 and OXM15E3 was 63-fold and 120-fold lower compared with OXM14 and OXM15, respectively (Figure 3D). Receptor binding affinity for the Glu-3 analogues at the rat GLP-1r was 2.7- to 4.4-fold greater than their Glu-3 counterparts (Figure 3D).

Substituting Glu at position 3 decreased peptide efficacy at the rat GCGr. The efficacy of OXM14E3, OXM15E3, and OXME3 was 200-, 25-, and 13-fold lower respectively compared with their Glu-3 equivalents OXM14, OXM15, and OXM. Glu-3 substitution increased rat GLP-1r efficacy by 1.2- to 1.6-fold (Figure 3D).

Concentrations of 0.03 to 1 nM OXM14E3 (Figure 3A) and 0.03 to 0.3 nM OXM15E3 (Figure 3B) decreased the percentage of glucagon-mediated cAMP production at the rat GCGr by a mean of 18.4% and 27.5%, respectively. Concentrations of 3 and 30 nM of OXM14E3 and OXM15E3 increased cAMP levels compared with glucagon stimulation alone (OXM14E3 30 nM: 155% increase, OXM15E3 30nM: 214% increase) (Figure 3A-B). Except 30 nM, all concentrations of OXME3 tested decreased glucagon-mediated cAMP accumulation by a mean of 40.3% (Figure 3C). A 30-nM dose of OXME3 increased cAMP levels by 9% compared with glucagon stimulation alone (Figure 3C).

## Discussion

Structurally, OXM is the 29 amino acids of glucagon with a C-terminal octapeptide tail.^2^ The dual agonist activity of OXM at the GCGr and GLP-1r makes it difficult to investigate the mechanisms involved in the weight-reducing effects of exogenous OXM administration because receptor antagonists also block the actions of endogenous glucagon and GLP-1. Previously it has been reported that exchanging the position-3 glutamine of OXM or a related analogue for Glu diminishes mouse GCGr activity without affecting activity at the mouse GLP-1r.^6^, ^7^ These GLP-1r selective Glu-3 peptides offer an alternative method for investigating the contribution of GCGr and GLP-1r activity to the metabolic effects of OXM and related analogues. Several previous studies have used Glu-3 analogues to explore the metabolic effects of OXM administration in mice, reporting that GCGr activity was necessary for,^6^, ^7^, ^8^ but the effect of Glu-3 peptide administration in rats has not been investigated.

The present study reports the novel observation that substituting Glu at position 3 of 2 OXM-like dual analogues caused a significant increase in body weight in a rat model, without altering food intake. In line with previous findings at the mouse receptor,^6^, ^7^ in vitro activity at the rat GCGr was diminished by Glu-3 substitution. Interestingly, glucagon-mediated activation of the rat GCGr in vitro was reduced by the lower concentrations of the Glu-3 analogues tested, including Glu-3 of native OXM. This suggests that the Glu-3 OXM analogues may be partial agonists that can also act as competitive antagonists in vitro, despite reduced rat GCGr binding affinity.

Glucagon administration increases energy expenditure^12^, ^13^ and in rodents this is postulated to be due to activation of brown adipose tissue.^14^ Exogenous peripheral administration of native OXM or long-acting analogues also increases energy expenditure^3^, ^4^, ^6^ and several murine studies have suggested a GCGr-mediated mechanism is responsible.^6^, ^7^ Glu-3 OXM analogues may antagonize the activity of endogenous glucagon, possibly causing a reduction in energy expenditure and resulting in the observed increase in rat body weight. However endogenous glucagon has not been shown to play a tonic role in energy expenditure or weight regulation. Investigation of the effects of Glu-3 peptides on energy expenditure in rats is needed.

The increase in body weight following Glu-3 administration may be a rat-specific phenomenon, as previous results with Glu-3 OXM-like peptides were obtained in mice and did not show an increase.^6^, ^7^ However the Glu-3 peptides used in these previous studies^6^, ^7^ were derivatized and administered at higher doses than in the present study. A recently published study^15^ demonstrated that analogues of glucose-dependent insulinotropic polypeptide (GIP) with a proline substitution at amino acid position 3 (Pro-3) were full agonists at the human GIP receptor and partial agonists that could acts as competitive antagonists at the rat and mouse GIP receptor. It is possible this may also be the case with Glu-3 OXM-like analogues. However, the agonist or antagonist properties of Glu-3 analogues at the mouse and human GCGrs have not been fully investigated. Additionally, differences in peptide and receptor homology between species may contribute to the differential effects observed with GIP analogues with a proline substitution at amino acid position 3. Human GIP has several different residues compared with rat and mouse GIP,^15^ whereas the amino acid sequences of glucagon and OXM are identical in the 3 species. Therefore the relevance of the findings with Pro-3 GIP analogues to the present study is unclear.

## Conclusions

OXM-like analogues with a Glu-3 substitution significantly increase body weight in a rat model, independent of food intake. Glu-3 analogues appear to be partial agonists at the rat GCGr and can also act as competitive antagonists in vitro. Therefore Glu-3 OXM-like analogues are unsuitable tools to investigate the effects of OXM analogue activity in a rat model.

## Conflicts of Interest

The authors have indicated that they have no conflicts of interest regarding the content of this article.
